# Asia-Pacific International Center of Excellence in Malaria Research: Maximizing Impact on Malaria Control Policy and Public Health in Cambodia and Papua New Guinea

**DOI:** 10.4269/ajtmh.21-1324

**Published:** 2022-10-13

**Authors:** Leanne J. Robinson, Moses Laman, Leo Makita, Dysoley Lek, Annie Dori, Rachael Farquhar, Amelie Vantaux, Benoit Witkowski, Stephan Karl, Ivo Mueller

**Affiliations:** ^1^ Papua New Guinea Institute of Medical Research, Madang, Papua New Guinea;; ^2^ Burnet Institute, Melbourne, Australia;; ^3^ Walter & Eliza Hall Institute of Medical Research, Melbourne, Australia;; ^4^ National Department of Health, Waigani, Papua New Guinea;; ^5^ National Centre for Parasitology, Entomology, and Malaria Control, Phnom Penh, Cambodia;; ^6^ School of Public Health, National Institute of Public Health, Phnom Penh, Cambodia;; ^7^ PNG Australia Transition to Health (PATH) Program, Waigani, Papua New Guinea;; ^8^ Malaria Molecular Epidemiology Unit, Institut Pasteur du Cambodge, Phnom Penh, Cambodia;; ^9^ Australian Institute of Tropical Health and Medicine, James Cook University, Smithfield, Australia

## Abstract

The Asia-Pacific International Center of Excellence in Malaria Research (ICEMR) was funded in 2016 to conduct a coordinated set of field and in-depth biological studies in Cambodia and Papua New Guinea (PNG), in sites that span the range of transmission intensities currently found in the Asia-Pacific regions. The overall objective is to gain an understanding of key parasite, human host, and vector factors involved in maintaining transmission in the face of intensified control and elimination programs, and to develop novel approaches to identify and target residual transmission foci. In this article, we will describe how the ICEMR program was designed to address key knowledge gaps and priority areas for the malaria control programs in each country. In PNG, partners have worked together on two consecutive ICEMR grants (2009–2016 and 2017–2024) and we present a case study of the partnership and engagement approach that has led to stronger coordination of research activities and integration with program, informing country-level strategic planning and prioritization of control activities. In both settings, the ICEMR program has generated insights into transmission foci, risk factors for ongoing transmission, highlighting the hidden burden of vivax malaria, and the need for additional complementary vector control tools. Finally, we will summarize the emerging research questions and priority areas—namely surveillance, vivax malaria, new vector control tools, and community/health systems-oriented approaches—where further tool development and implementation research have been identified as being needed to guide policy.

## KEY PRIORITIES OF MOH IN CAMBODIA AND PNG AT THE START OF THE PROGRAM

At the 2015 East Asia Summit, the Asia-Pacific heads of state pledged to work toward a malaria-free Asia Pacific by 2030.^1^ Although impressive reductions in malaria have been achieved across the entire region in the last 20 years, this is still a highly ambitious and challenging goal, technically, financially, and politically. It will require leadership, evidence-based and adaptive policy-making, and a highly collaborative intersectoral approach.

The current status of malaria in the Asia-Pacific ranges from countries that have been declared malaria-free (e.g., Singapore, Sri Lanka, and most recently, China), those that are actively attempting country-wide elimination (e.g., Korea, Malaysia, Thailand, and Cambodia) to countries with moderate to high transmission that are still in the control phase (e.g., Papua New Guinea [PNG] or [Eastern] Indonesia).^2^ Despite the great variation in their respective malaria epidemiology and health system capacity, Asia-Pacific countries face similar challenges in their quest for malaria elimination: 1) the fragmentation of malaria transmission into residual pockets of high transmission surround by areas with no or little transmission; 2) the increasing proportion of *Plasmodium vivax* infections; 3) the high burden of asymptomatic infections that are not detected by health services that are largely focused on diagnosis and treatment of clinical cases; 4) presence or threat of the spreading multidrug resistance including artemisinin and partner drug resistance;^3^ and 5) presence or threat of spreading insecticide resistance and behavioral resilience of mosquito populations to vector control. Given these common challenges despite different average transmission intensities and progress toward elimination, Cambodia and PNG were selected as the focus counties of the 2017–2023 Asia-Pacific International Center of Excellence in Malaria Research (ICEMR). In both setting, the National Malaria Control Program (NMCP), CNM in Cambodia and NMCP in PNG, are key members of the collaborative ICEMR program and play an important role in ensuring the prioritization of objectives that will provide science-based evidence addressing the critical knowledge gaps about malaria transmission and the effectiveness of existing strategies in each setting.

### Cambodia.

Over the past 15 years, Cambodia has successfully reduced the burden of malaria from a peak of more than 100,000 cases or 7.4 per 1,000 population in 2006 to 62,000 cases or 3.9 per 1,000 population in 2018.^4^ Malaria-related mortality also declined from 400 per year to 0 over the same period. The explicit goals and objectives of the Cambodia National Strategic Plan 2011–2025 included a vision to ensure partial elimination of malaria by 2020 (focus on *Plasmodium falciparum*) and total elimination by 2025 (focus on *P. vivax*).^4^ Based upon this strategic plan and observed success in strengthening the health system with village malaria workers to enable universal access to timely malaria diagnosis and treatment, the government developed the Cambodia Malaria Elimination Action Framework 2016–2020 to guide their work with local and international partners on malaria elimination.^4^

In Cambodia, CNM coordinates all vector borne and parasitic disease program activities and makes appropriate policy, program, and operational recommendations. It works closely with partners to conduct operational research studies, the outcomes of which directly feed back into program enhancement. Given the complex operating environment, with numerous academic and NGO stakeholders working to support the elimination efforts, consultation with the program identified two key priorities for the ICEMR program: 1) In-depth studies on forest malaria and evidence to improve future surveillance strategies to identify high-risk populations and locate malaria foci; and 2) generating evidence to guide strategies to address the issue of persistent vivax malaria.

### Papua New Guinea.

Over the past 15 years, PNG has made substantial progress in malaria control. With financial support from The Global Fund to Fight AIDS, Tuberculosis, and Malaria (The Global Fund), 3-yearly distribution campaigns have provided long-lasting insecticidal nets (LLIN) to households since 2004 and malaria rapid diagnostic tests (mRDT) and artemisinin-based combination therapy (ACT) have been scaled up at health facilities throughout the country since late 2011.^5^^,^^6^ In selected areas of the country, home-based management of malaria programs were implemented and behavior change campaigns supported the roll-out of preventative and curative interventions. Over this same period, nationwide malaria indicator surveys documented a decreasing malaria infection prevalence by light microscopy (LM) from 11% in 2008/2009 to < 1% in 2013/14.^7^ In-depth community surveys conducted as part of the 2009–2016 Southwest Pacific ICEMR, confirmed reductions in the prevalence of qPCR-detectable infections from 55% to 6%.^8^^,^^9^ However, by 2016/2017 a number of factors combined to reinforce the fragility of control in malaria-endemic settings. The nationwide prevalence by LM had increased to 9.5%^10^ and the qPCR prevalence in North Coast areas had to 41%.^11^^,^^12^ Likely factors included reduced Global Fund and PNG government support to the program contributed limiting the availability of RDTs and ACTs across PNG, the reduced bioefficacy of bed nets distributed after 2013 in PNG^13^ and changes in mosquito biting behavior (from the night to early evening) reducing the effectiveness of bed nets.^14^

The vision of the National Malaria Strategic Plan (2021–2025) is “A malaria-free Papua New Guinea by 2030” with short-term goals (2019–2025) to reduce morbidity by 63% and mortality by 90% and to eliminate malaria in the Autonomous Region of Bougainville. The longer-term goal is the elimination of malaria (2025 onwards), when existing and new tools in combination with strengthened health systems will make national elimination feasible.^15^

The key knowledge gaps and research priorities identified during the 2009–2016 Southwest Pacific ICEMR to be addressed in addressed in the 2017–2024 Asia-Pacific ICEMR were: 1) to better understand transmission, key drivers and the emergence and spread of drug resistance to inform the implementation of improved surveillance strategies; 2) to lead evidence-based coordination and monitoring of the efficacy of vector control tools; and 3) To refine malaria transmission models based on local data to predict impact of revised surveillance strategies and vector control tools.

Boxes 1 and 2 outline the different priorities in Cambodia and PNG and summarize the approaches undertaken to address them. A detailed description of the scientific program of studies developed to address these critical questions is provided in the article by Mueller et al. in this issue. These interconnected, multidisciplinary studies aim to identify and characterize critical parasite, vector, and host features that contribute to continued transmission despite intensive national malaria control and elimination programs. This integrated, holistic approach to understanding key malaria risk areas and populations, key risk factors for the acquisition of infections, and the dynamics and range of spread of parasite populations is crucial to eliminating malaria from the Asia-Pacific region.

**Box 1 – Cambodia**

Major progress towards pre-elimination for *P. falciparum* but drug resistance and *P. vivax* remain major challenges.

*Priorities:* Understanding forest transmission, improving targeted surveillance and intervention strategies, and generating evidence to prioritise and address the issue of persistent vivax malaria.

*Approaches:*

1) Population studies investigating the extent and nature of spatial and temporal heterogeneity of malaria transmission
2) Studies of mosquito vector ecology, behaviour and risk factors for human-vector contact
3) Applying immunological and genomic assays to understand parasite and host interactions.

Photo: *Double-net traps set up in a forest site in Kaev Seima District, Mondulkiri Province, Cambodia. Credit: A. Vantaux, Institut Pasteur.* 
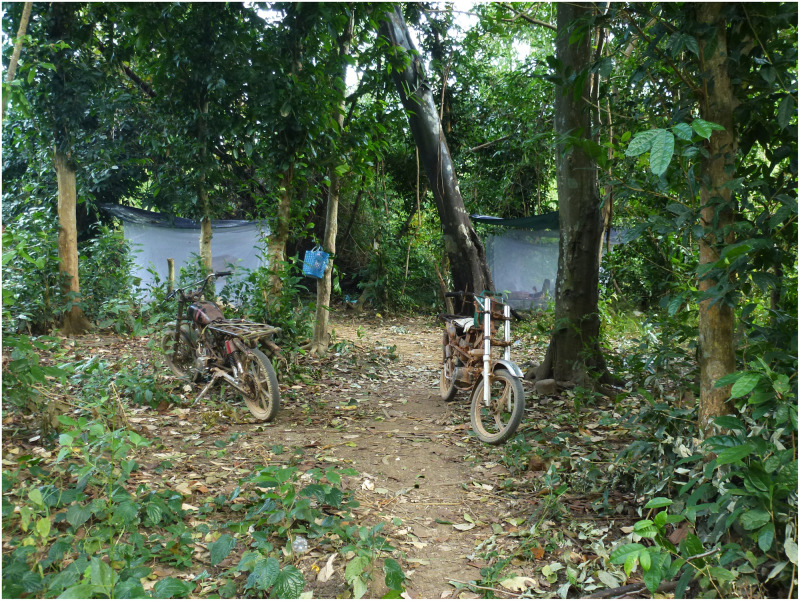

**Box 2 – Papua New Guinea**

Higher malaria burden, transitioning towards stratification and tailored sub-national control, high burden of *P. vivax* and emergence of drug resistance markers.

*Priorities:* Understanding transmission heterogeneity, the parasite, mosquito and human factors driving it, to inform revised surveillance and intervention strategies.

Approaches:

1) Population surveys and longitudinal cohorts to understand transmission, key drivers and the emergence and spread of drug resistance;
2) Studies of mosquito abundance, behaviour and efficacy of control tools;
3) Applying immunological and genomic assays to understand parasite and host interactions;
4) Refining malaria transmission models based on local data in order to predict the impact new strategies.

Photo: *PNGIMR ICEMR Epidemiology team enrolling study participants. Photo credit: L. Robinson.* 
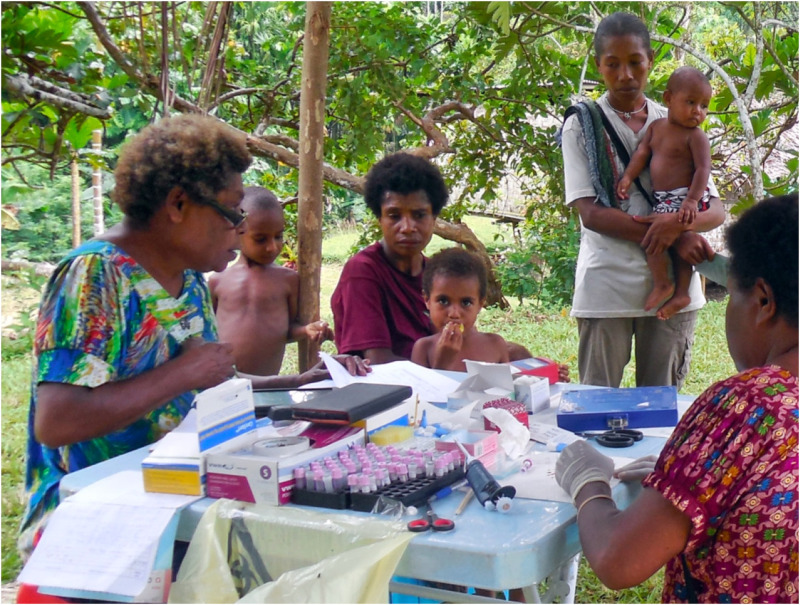

## CASE STUDY: PNG NATIONAL MALARIA CONTROL PROGRAM PARTNERSHIP AND ENGAGEMENT APPROACH

In PNG, where fiscal constraints and limited resources pose challenges to the NMCP it is critical to recognize that coordination, local ownership, sharing of resources, and a genuine partnership-way of working is essential for research to contribute toward evidence-based policy and sustainable systemic change. In 2010, the NMCP commenced a technical working group (TWG) bringing together key stakeholders involved in malaria activities in PNG.^16^ This TWG has been crucial in reviewing science-based evidence generated through the ICEMR project and has initiated the exploration of other technical areas that were seen as priority areas after the review of evidence, under Malaria Strategic Plans.

More recently, to ensure strengthened coordination and engagement with partners, the NMCPs partnership management unit (PMU) provides explicit partnership brokering support to the program and its partners from research institutions, government bodies, universities, and private sector donors and implementers.

The ICEMR is committed to empower the program and with PMU support, the NMCP has been able to use research findings from the ICEMR project to identify gaps and strengthen collaboration to enable the expansion of implementation activities established on the foundation of science-based evidence.

For example, in alignment with this partnership approach, scientific evidence generated through the ICEMR program has provided a platform for the NMCP to identify various research gaps and seek specific funding support for programs to address additional vector control and strengthened vivax case management.

Due to the solid foundation of engagement, there is mutual awareness of ICEMR project activities NMCP program activities that is highly beneficial to overcome operational challenges in the field in a timely manner. By working hand-in-hand together with the support and contribution from multiple partners, when barriers and bottlenecks arise, the NMCP and ICEMR teams are able to leverage resources from across multiple networks and find innovative ways forward.

On a regional level, the NMCP engages with different stakeholders to ensure that malaria work in country aligns with regional priorities as well as national and provincial level strategies. The way of working adopted by the NMCP recognizes the broader social and cost-benefit of partnerships and ensures that local ownership and a shared vision are incorporated into all research activities to strengthen efforts toward sustainable systemic change.

## IMPACT OF ICEMR ON MALARIA POLICY AND PUBLIC HEALTH

### Insights into forest malaria transmission in Cambodia.

In Cambodia, transmission risk is associated with forest-related activities. Epidemiological and molecular data from a community cross-sectional study revealed that 8.3% of inhabitants in Kaev Seima are infected with *Plasmodium spp.* parasites, with 68% of infections due to *P. vivax*. Prevalence was found to range widely across villages with a trend toward very high levels of infections in villages inside the forest, where all inhabitants were at risk of malaria infection. In villages outside the forest or on the fringe, the risk of infection was highly associated with work in and travel to the forest and highest in working age men. The vast majority of infections (96%) were asymptomatic and/or undetectable by standard microscopy or rapid diagnostic tests. In parallel with these epidemiological studies, intensive vector studies have revealed an entomological inoculation rate (number of infectious bites per person per unit of time) that is six times higher in the forest compared with the other sampled sites and a daytime biting rate of approximately 20% with infectious mosquitoes collected both during day and night.

Altogether, these results strengthen the hypothesis that the forests are the main risk areas for human malaria transmission in Cambodia and highlight the importance of daytime biting behavior of mosquitoes as a potential source for transmission. The data reinforced CNM program planning that targeting at risk groups based in forest proximity may be more cost effective than population-wide prevention strategies. Our results also highlight the high burden of mostly asymptomatic *P. vivax* infections and the importance of specific control efforts aimed at this parasite. The only licensed drug with hypnozoiticidal effect for the treatment of *P. vivax* infections is primaquine, which is part of the national treatment guidelines but not yet implemented because of concerns over the drug’s safety in patients with glucose-6-phosphate dehydrogenase (G6PD) deficiency. CNM have progressed with panning implementation of G6PD point-of-care testing and radical cure treatment with primaquine to specifically address the *P. vivax* burden and have also acknowledged promising new advances in the use of multiple *P. vivax* antigens to detect antibodies as serological markers for recent exposure as a proxy for identifying potential hypnozoite carriers developed as part of the ICEMR.^17^

### Understanding the epidemiology and drivers of ongoing transmission in Papua New Guinea.

In PNG, where transmission is largely peri-domestic and 90% of the population live in rural villages, the focus has been on understanding the changing epidemiology of transmission because of scale of control interventions and the drivers of ongoing transmission through integrated parasite, vector, and human behavioral studies.

In communities of East Sepik Province, the prevalence of *P. falciparum* decreased from 55% (2005) to 9% (2013) and *P. vivax* from 36 to 6%.^8^ The reductions led to a more equal distribution of infections and illness across age groups, but increased spatial heterogeneity, that is, larger differences among villages and among households within villages. As transmission decreased, malaria risk became more stratified and localized hotspots were observed within villages and households.^8^^,^^11^ The high heterogeneity of malaria in 2013 highlighted the importance of surveillance and targeted interventions to accelerate toward elimination. By understanding the village and household-level spatial heterogeneity for malaria risk and the utility of key malaria indicators for the identification of hotspots of malaria transmission at village-level, ICEMR studies are supporting the program to optimize malaria surveillance and response.^11^

By comparing three longitudinal child cohorts before (2006/2007), during (2008), and after scale-up of control interventions (2013), we demonstrated that intensified vector control and routine case management had a greater initial impact on rates of *P. falciparum* compared with *P. vivax* infections but equal impact on *P. falciparum* and *P. vivax* clinical malaria episodes in young children.^18^ This data highlighted how standard malaria control tools effectively reduce new mosquito-derived infections for both species but have a delayed impact on *P. vivax* due to the reservoir of previously acquired hypnozoites. Strengthening the implementation of *P. vivax* radical cure was subsequently clearly prioritized in the PNG NMCP strategy for 2021–2025 and is the focus of a major program of implementation research that NMCP is supporting.

Health facility clinical surveillance data is also being linked with in-depth community prevalence/incidence and spatial mapping data from a recently completed longitudinal cohort study to validate routinely collected indicators for their ability to identify high-risk areas or groups of people. In addition, there has been continued surveillance of severe pediatric malaria, which has documented on overall downward trend in severe malaria admissions and severe malaria case fatality in the 2015–2020 period compared with the 2007–2009 period (unpublished data, Laman & Lufele). There has also been an upward shift in the median age of presentation between these two periods of surveillance. The severe malaria age shift parallels the declining burden of malaria over this period and highlights the importance of the long-term ICEMR surveillance. This would not have been captured in studies that run for a shorter period of time. As part of this finding, the PNG Pediatric Society has been informed in its recent annual meeting with the aim of creating clinical awareness about this changing trend and to protect the age group at risk of severe malaria.

Long-lasting insecticidal nets are the only vector control tool implemented by the NMCP in PNG on a nationwide level. Between 2010 and 2019, 12.8 million LLINs were delivered to the country, with approximately 10.2 million since 2013. In close collaboration with NMCP, the ICEMR tested the bioefficacy of LLINs manufactured between 2013 and 2019 collected from villages and LLIN distributors. Reduced bioefficacy was observed, with only 16% of LLINs manufactured after 2013 fulfilling the required WHO bioefficacy standards.^13^ In contrast, all LLINs manufactured prior to 2013 met the WHO bioefficacy standards. These results directly informed the program’s procurement of nets and strengthened the in-country quality control testing of nets.

The simultaneous collection of parasite, mosquito, and human behavioral data was in the ICEMR community cross-sectional and cohort studies provides unique insights into the drivers of ongoing transmission. The peak biting time of the predominant Anopheline vector in study villages has been observed to correspond with a time when 20–70% of people in that village are not yet indoors and underneath their LLINs, highlighting the need for additional tools to complement LLINS, and target outdoor biting. These findings have directly informed the NMCP and plans to expand the range of vector control tools available within PNG is a priority of the 2021–2025 NSP.

## IMPORTANCE OF EMBEDDING POLICY-RELEVANT PRIORITIES WITHIN SCIENTIFIC OR DISCOVERY-BASED RESEARCH

In PNG, the time required for full policy change can be between 2 and 3 years and is a collaborative effort among the TWG, technical stakeholders’ group, NMCP program manager, and Senior Executive management team.^16^ Similarly, in Cambodia, multiple TWGs, the Ministry of Health and WHO are involved and 6–12 months is typically required to change antimalarial policy.^16^ In Cambodia, antimalarial policy is reviewed more regularly compared with other diseases due to the speed with which drug resistance has evolved and the requirement to be responsive to this. In both settings, it is, therefore, critical that scientific research questions are codeveloped with members of the NMCP TWG and CNM to prioritize the generation of evidence required by policy-makers and minimize the time between evidence generation and policy change. In PNG, the ICEMR program provided critical insights into the high proportion of asymptomatic low-density infections that were persisting in key areas at a time when national level surveillance indicated prevalence was < 1% by LM.^9^ This enabled NMCP to prioritize the inclusion of molecular surveillance in their National Malaria Strategic Plan and future monitoring and evaluation activities. As mentioned above, PNGIMR’s work with NMCP and RAM to document the reduced bioefficacy of LLINs distributed since 2013 also directly influenced LLIN procurement and distributions in PNG, as well as strengthening the in-country quality control program.

Although, in PNG, the program was developed from a research lens, the partnership-based approach has meant, principles such as inclusivity, transparency, mutual benefit, and a shared vision have remained central to all decision-making along the way. For the teams in both settings, it has been critical for key stakeholders to be involved in annual SAG meetings among other country ICEMR programs of work, to provide a platform for continued discussion around country priorities, and research activities. Fostering a collaborative environment between researchers and policy-makers has allowed space for rigorous scientific studies to generate meaningful evidence that has directly shaped CNM’s priorities, informed the PNG NMCP TWG on implementation research activities and subsequently paved the way to policy change. The collaborative environment created through the partnership-based approach also led to additional resourcing and implementation studies necessary to address gaps identified in the ICEMR program.

## NEW AND EMERGING NEEDS AND RESEARCH QUESTIONS

The ICEMR program of work has been an opportunity to strengthen new and ongoing malaria activities and research programs in PNG and Cambodia by innovative research approaches with programmatic priorities and codeveloping research questions as new challenges and areas of interest emerge. There are several key themes that have emerged over the past 5 years, which will both be a key focus for the remaining 2 years of our program and are providing the opportunity for partners to leverage additional funding for priority programs of research and implementation.

### Transforming surveillance.

In Cambodia, strategies targeting occupational risk groups in villages outside the forest have emerged as priority programmatic considerations to eliminate transmission in these areas. Whereas the generally elevated risk in village populations near or inside the forest has highlighted the potential suitability of population-oriented interventions, such as mass drug administration or test and treat programs, with sensitive molecular or serological diagnostics. Clearly, the hopes that “highly sensitive” field deployable diagnostic tests might provide the opportunity for effective screening and treatment or reactive case management programs have not yet been realized. Given that forest work has emerged as one of the strongest risk factors for becoming infected with falciparum malaria in Cambodia, novel approaches to provide forest goers with malaria prophylaxis and/or additional personal mosquito protection tools are major areas that CNM would like to further explore. Similarly, given that there is a clear need to target additional intervention toward the hidden reservoir of vivax infections, there is a new program of work piloting the implementation of *P. vivax* serological testing and treatment in several villages in Mondulkiri Province and the ICEMR will support cross-sectional surveys and acceptability studies to measure the impact and community attitudes toward this new vivax-specific intervention.

In PNG, one of the most pressing needs that has emerged in the past 5 years is to strengthen the surveillance of antimalarial drug resistance mutations. Kelch13 C580Y mutations associated with artemisinin resistance were discovered for the first time in PNG in 2016 in *P. falciparum* isolates collected from Wewak, East Sepik Province.^19^ Although the genomic data was consistent with kelch 13 C580Y emerging in the local parasite population, it wasn’t clear whether it emerged in PNG, West Papua, or another nearby location.^20^ Establishing K13 genotyping assays at PNGIMR has therefore been a major focus of several NMCP and PNGIMR led programs of work and tools developed by the Genomics Core of the Asia-Pacific ICEMR have supported this. In addition, monitoring the evolution and spread of K13 mutations has now become a priority goal of the Asia-Pacific ICEMR program, providing opportunities for technical transfer of knowledge and skills between Cambodia and PNG. PNGIMR and NMCP also successfully obtained independent funding for a Gates Grand Challenges Program of work to consider new approaches for integrating molecular surveillance into malaria control programs.

### Vector control.

Entomological data from PNG on early, outdoor mosquito biting, plasticity in host selection, and preference of habitat has strengthened the case for introducing additional vector control tools in the country. Recognizing the LLINs alone will not lead to malaria elimination; the ICEMR Program has positioned the NMCP to apply for additional funding from international donors to investigate additional vector control tools such as Spatial Repellents, Indoor Residual Spraying, and Larval Source Management to efficiently reduce human vector contact across different settings. Increased vector control strategies and corresponding research, now incorporated into the National Malaria Strategic Plan for 2021–2025, is an emerging priority area for researchers and the program to generate evidence. The ICEMR program has created a solid foundation of evidence on the longitudinal epidemiological and entomological malaria trends in four villages across the North Coast of Madang, which is currently allowing implementation research on the impact of IRS to be undertaken to directly inform the programmatic scale-up throughout

### Vivax malaria.

In both settings, data on the burden of vivax infections from ICEMR studies has highlighted that the implementation of effective strategies to control vivax malaria is essential for accelerating toward the 2030 timeline for malaria elimination in the region. In Cambodia, there is a focus on integrating effective radical cure with standard case management and ongoing targeted elimination activities. There is also interest to explore the efficacy and acceptability of novel vivax-specific public health interventions, such as serological testing and treatment (*Pv*SeroTAT), which provide an opportunity to identify and target the hypnozoite burden.^21^ Although liver-stage hypnozoites are unable to be detected directly, serological responses to *P. vivax* blood-stage infections can be used as highly-sensitive biomarkers of the hidden parasite reservoir.^17^ In PNG, the NMCP and PNGIMR are leading a program of implementation research that will generate evidence on the feasibility and cost-effectiveness of point-of-care quantitative G6PD testing prior to shorter course high-dose primaquine that will directly inform revised treatment guidelines, implementation, and scale-up of *P. vivax* management and radical cure.

### Understanding communities and health service providers to maximize impact.

The ICEMR program has been critical in understanding the parasite and vector drivers of transmission and also highlighted a need to further understand community social and cultural patterns, livelihoods, and response to specific interventions that contribute to the malaria burden across the country.^22^ Knowledge, perceptions, and practices related to malaria transmission and health systems acceptability of new malaria interventions is now seen as a key component of all NMCP research programs and embedded into future implementation research and programmatic activities. Tailored qualitative ethnographic methodology, such as interviews, structured observations, photovoice, and focus group discussions, are critical research techniques to complement and contextualize the quantitative scientific based evidence generated through the ICEMR program and future NMCP lead activities.

## CONCLUSION

Our ICEMR program has provided significant new evidence and guidance to the malaria control programs in Cambodia and PNG, addressing current key knowledge gaps and highlighting new and emerging research questions that will need to be addressed to eliminate malaria. Our ICEMR program has sought close engagement and exchange with the country’s malaria control programs, enabling them to identify key priority areas. Our integrated and holistic approaches have resulted in tangible changes and influences in the areas of malaria surveillance, vector control, diagnosis, and treatment of *P. vivax*, and the recognition of the importance of qualitative research to understand community and health systems perspectives.
